# A nomogram for predicting the likelihood of lymph node metastasis in early gastric signet ring cell carcinoma: A single center retrospective analysis with external validation

**DOI:** 10.1097/MD.0000000000005393

**Published:** 2016-11-18

**Authors:** Chun Guang Guo, Yan Jia Chen, Hu Ren, Hong Zhou, Ju Fang Shi, Xing Hua Yuan, Ping Zhao, Dong Bing Zhao, Gui Qi Wang

**Affiliations:** a Department of Abdominal Surgical Oncology, National Cancer Center/Cancer Hospital; b Medical Statistics Office; c Program Office for Cancer Screening in Urban China; d Department of Endoscopy, National Cancer Center/Cancer Hospital, Chinese Academy of Medical Sciences and Peking Union Medical College, Beijing, China.

**Keywords:** early gastric cancer, endoscopic submucosal dissection, lymph node metastasis, nomogram, signet ring cell carcinoma

## Abstract

Treatment algorithm has not been established for early gastric cancer with signet ring cell carcinoma (SRC), which has a reported low rate of lymph node metastasis (LNM) similar to differentiated cancer. A cohort of 256 patients with early gastric SRC at our center between January 2002 and December 2015 were retrospectively reviewed. Multivariate logistic regression analysis was used to determine the independent factors of LNM. A nomogram for predicting LNM was constructed and internally validated. Additional external validation was performed using the database from Cancer Institute Ariake Hospital in Tokyo (n = 1273). Clinical performance of the model was assessed by decision analysis of curve. The overall LNM incidence was 12.9% (33/256). The multivariate logistic model identified sex, tumor size, and LVI as covariates associated with LNM. Subsequently, a nomogram consisted of sex, tumor size, and depth of invasion was established. The model showed qualified discrimination ability both in internal validation (area under curve, 0.801; 95% confidence interval [CI], 0.729–0.873) and in external dataset (area under curve, 0.707; 95% CI, 0.657–0.758). Based on the nomogram, treatment algorithm for early gastric SRC was proposed to assist clinicians in making better decisions. We developed a nomogram predicting risk of LNM for early gastric SRC, which should be helpful for patient counseling and surgical decision-making.

## 1. Introduction

Early gastric cancer (EGC) with signet ring cell carcinoma (SRC) was reported to have a favorable outcome, with a 5-year survival rate of more than 90%.^[1,2]^ Although categorized as undifferentiated histology, early gastric SRC has a low rate of lymph node metastasis (LNM) similar to the differentiated cancer.^[3–5]^ Several studies revealed no LNM when SRC lesions were confined to mucosa, without lymphovascular invasion (LVI), and less than 15^[6]^ or 20 mm.^[7]^ Recently, Pyo et al^[8]^ introduced a risk-scoring tool for gastric mucosal SRC. Patients were scored according to 3 variables, including tumor size, macroscopic type, and LVI. Of all patients scored with zero, only 1.1% was involved with LNM and a regular surveillance is suggested. Consequently, endoscopic submucosal dissection (ESD) has been indicated for early gastric SRC to maintain the quality of life in East Asia.^[6,9]^ However, most studies only focused on identifying predictive factors associated with LNM, and failed to provide a quantified risk of LNM for individuals.^[6–10]^ Moreover, the clinical performance between different treatment strategies has not been assessed yet.

Nomogram is a user-friendly graphic tool for predicting probability of event, which incorporates several associated factors based on a statistical procedure. As an easy-to-use and advanced tool for personalized treatment, nomogram has been widely applied for clinical decision-making in oncology research, such as breast cancer and prostate cancer.^[11,12]^ One of the primary advantages of nomograms is the ability to estimate risk on the basis of individual and disease characteristics, which could help clinicians identify patients who might derive more benefits from an appropriate treatment.^[13]^

In this study, we will investigate the predictive factors of LNM in early gastric SRC. Moreover, we aimed to build a treatment algorithm for early gastric SRC by establishing and validating a nomogram for LNM prediction.

## 2. Materials and methods

Medical records of 1676 patients who underwent consecutively curative gastrectomy for early gastric adenocarcinoma at Cancer Hospital, Chinese Academy of Medical Sciences (CAMS) in Beijing, China, between 2002 and 2015, were reviewed retrospectively. Patients who have stump gastric cancer; neoadjuvant chemotherapy; incomplete information; multiple lesions, and combined with other malignancies were excluded. This study received institutional review board approval. A cohort of 1273 patients with early gastric SRC diagnosed at Cancer Institute Ariake Hospital, Tokyo, in Japan, between 1946 and 2007, were eligible for aforementioned inclusion criteria and selected as validation set with the investigators’ approval.^[14]^

All clinicopathological variables were retrieved from a prospective database, including sex, age at diagnosis, tumor location, tumor size, macroscopic type, depth of invasion, number of lymph node, positive lymph node, and LVI. The macroscopic appearance of tumor was classified by Japanese Classification of Gastric Cancer, such as I type (elevated), II type (superficial), and III type (depressed).^[15]^ According to invasion depth, lesions are categorized as mucosal cancer (T1a) or submucosa cancer (T1b). All harvested lymph nodes were examined by spiting in half along the maximum diameter and stained with H&E section. Tumor invasion and N staging were defined in accordance with the American Joint Committee on Cancer staging.^[16]^ Two experienced pathologists reviewed all pathological slices.

Descriptive data are presented as mean ± SD. For comparisons between different groups, continuous variables are analyzed using the Student *t* test, and categorical variables were analyzed using chi-square test. Factors significant in univariate analysis are included in logistic regression analysis to identify independent variables. Nomogram was developed as the procedure described by Iasonos et al.^[17]^ The discrimination power of the nomogram was evaluated by concordance index, which is identical to the area under the receiver operating characteristic curve. The area under curve (AUC) ranges from 0 to 1, with 1 indicating perfect concordance, 0.5 indicating no better concordance than chance. Subsequently we constructed a plot of calibration, which was internally and externally validated with 500 bootstrap repetitions to reduce the overfit bias. Finally, a decision analysis of curve was performed to evaluate the clinical utility and identify optimal threshold range by quantifying the net benefits.^[18]^ The Statistical Package for the Social Sciences (SPSS) for Windows, Version 18.0 (SPSS Inc., Chicago, IL) or the rms package (version 4.4-2) and pROC package (version 1.8) in R version 3.2.2 were used in this study.^[19]^*P* values were 2 sided, and values of less than 0.05 were considered statistically significant.

## 3. Results

### 3.1. Demographics of patients in training set and validation set

Total of 256 patients with early gastric SRC who underwent curative resection at CAMS were analyzed as training set. Table 1 listed all patient demographics. Ratio of male: female was equal, and age at diagnosis was 50.0 ± 11.8 years (range from 24 to 82). Most lesions located in lower third (217, 84.8%), and the remaining in middle (27, 10.5%), upper (4, 1.6%), and entire (8, 3.1%). Numbers of I type, II type, and III type were 30 (11.7%), 66 (25.8%), and 160 (62.5%), respectively. Tumor size was 2.64 ± 1.54 cm. LNM was revealed in 12.9% of patients (33/256). Mucosal cancer and submucosal cancer account for 69.9% (179/256) and 30.1% (77/256), respectively. LVI was found in 15 patents (5.9%).

**Table 1:**
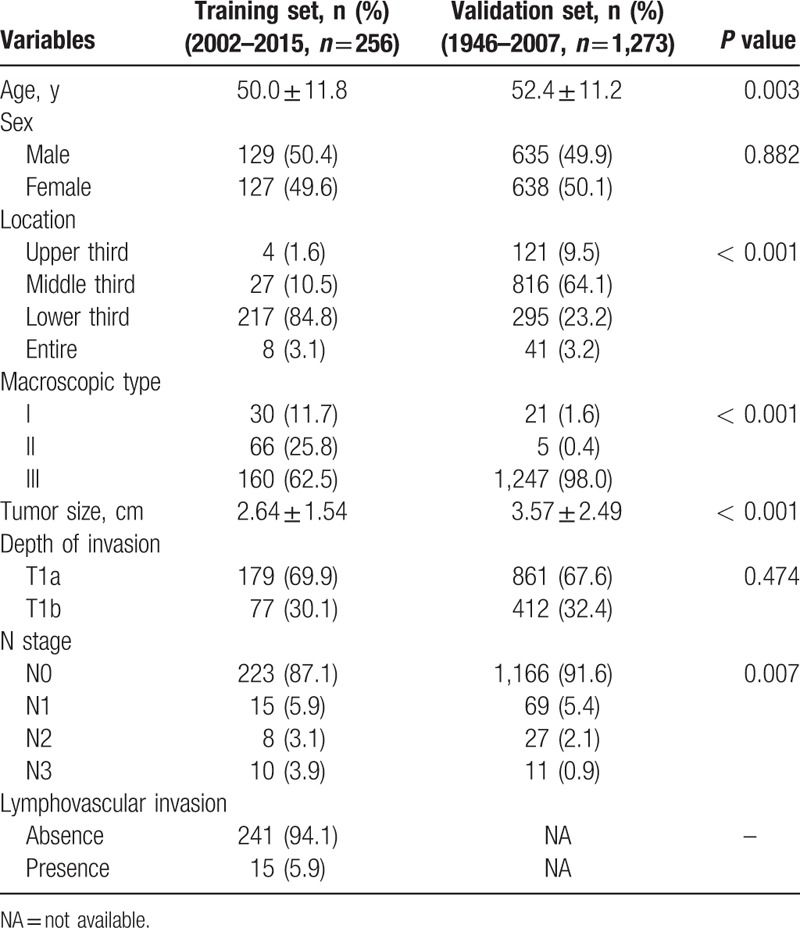
Demographics of patients with early gastric cancer with signet ring cell carcinoma in training set and validation set.

Clinicopathological characteristics of 1273 patients with early gastric SRC diagnosed at Cancer Institute Ariake Hospital were listed in Table 1. Except sex (*P* = 0.882) and depth of invasion (*P* = 0.474), there was significant difference in age at diagnosis (*P* = 0.003), tumor location (*P* < 0.001), macroscopic type (*P* < 0.001), tumor size (*P* < 0.001), and N stage (*P* = 0.007) between 2 datasets.

### 3.2. Risk factors associated with LNM for early gastric SRC

Continuous variables (age and size) were examined using restricted cubic splines. Four factors, including sex (*P* = 0.013), tumor size (*P* < 0.001), depth of invasion (*P* = 0.013), and LVI (*P* < 0.001) are confirmed significantly associated with LNM in univariate analysis. Through multivariate analysis, size more than 3 cm (odds ratio [OR] = 12.790, 95% confidence interval [CI], 3.452–47.392; *P* < 0.001), female sex (OR = 2.675, 95% CI, 1.118–6.402; *P* = 0.027) and presence of LVI (OR = 6.564, 95% CI, 1.719–25.060; *P* = 0.006) were confirmed as independent risk factors for LNM, whereas there was no significant difference between tumor depth and LNM (*P* = 0.531) (Table 2).

**Table 2:**
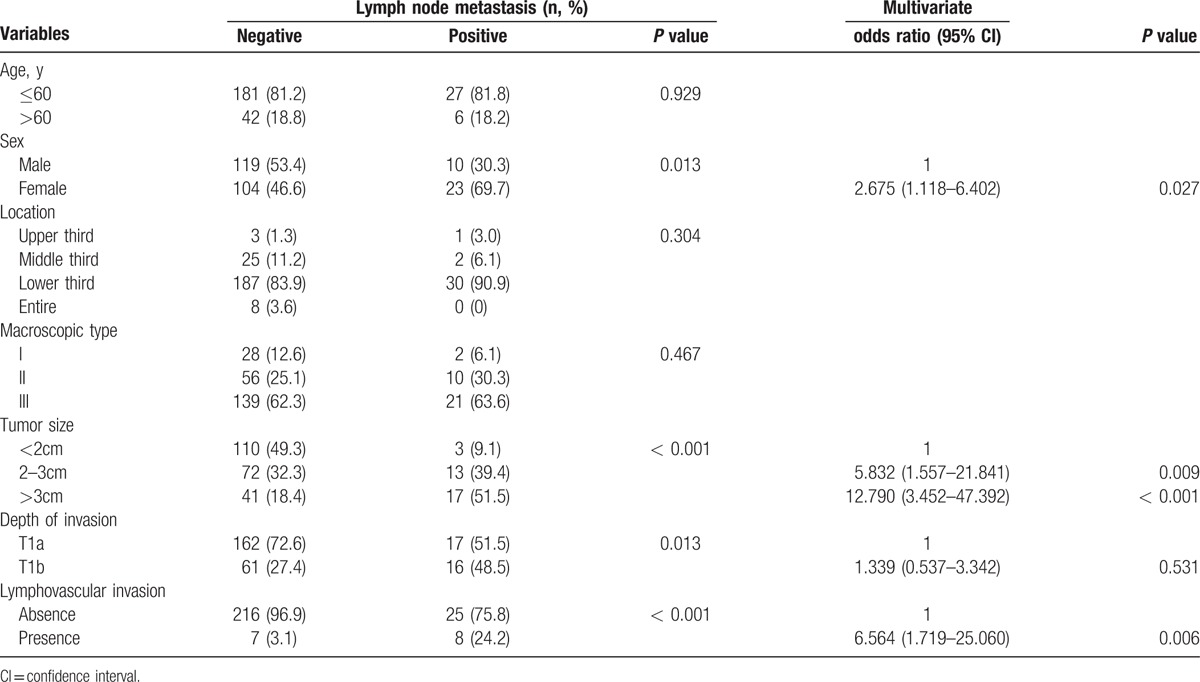
Univariate and multivariate analyses of factors associated with lymph node metastasis in training set.

As impossible to accurately confirm status of LVI before surgery, relationship between clinicopathological factors and LVI was assessed. Tumor size larger than 3 cm (OR = 4.432, 95% CI, 1.006–19.520; *P* = 0.049) and T1b (OR = 38.255, 95% CI, 4.883–299.668; *P* = 0.001) were significantly associated with LVI by multivariate analysis (Table 3). Consequently, depth of invasion was incorporated into the prediction model.

**Table 3:**
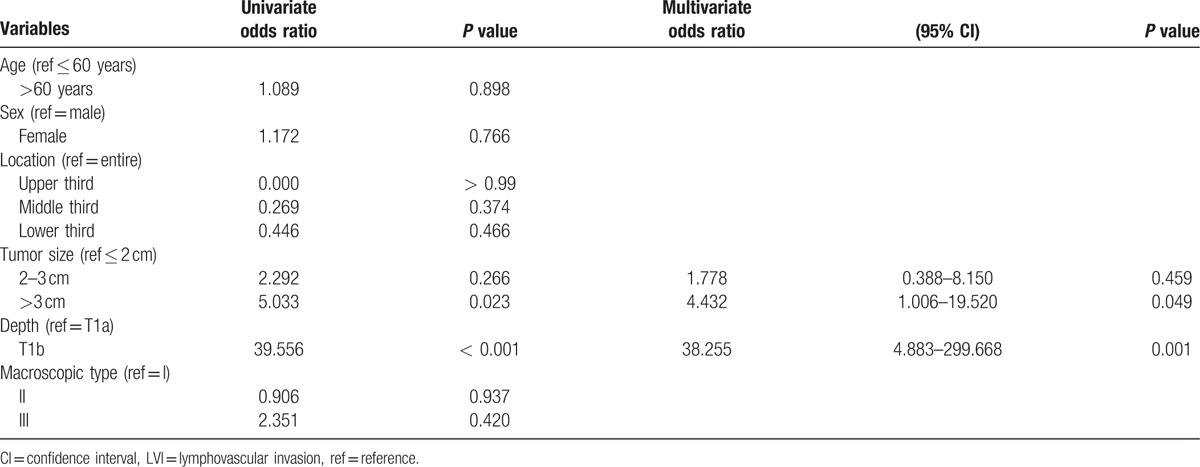
Relationship between clinicopathological factors and LVI in training set.

### 3.3. Establishment and validation of a nomogram for predicting LNM in early gastric SRC

**Figure 1:**
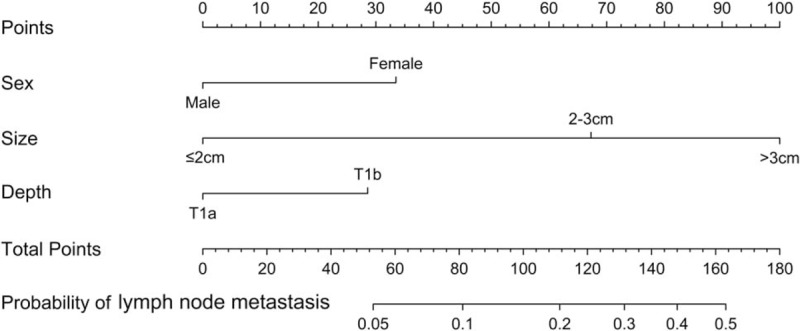
A nomogram predicting lymph node metastasis for early gastric cancer with signet ring cell carcinoma. Each level within variables was assigned a score according to the point scale. By summing up the total score and locating it on the total point scale, a corresponding probability of lymph node metastasis for each individual was determined.

A nomogram predicting risk of LNM was established on the basis of the multivariate logistic regression model. Tumor size was the largest contributor to the score, and then followed by sex and depth of invasion in Fig. 1.

**Figure 2:**
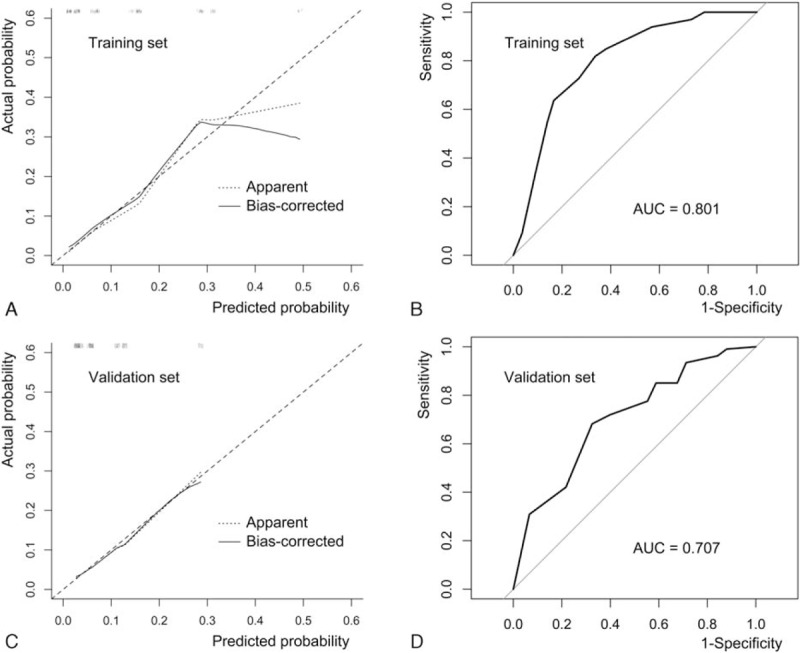
Validation of nomogram in training set and validation set. (A) Calibration plot of nomogram in training set. After 500 repetitions of bootstrap, the bias-corrected plot showed a good agreement between the predicted probability and actual probability (Mean absolute error = 0.021). (B) The AUC of nomogram in training set was 0.801 (95% CI, 0.729–0.873) after 500 repetitions of bootstrap (Delong). (C) Calibration plot of nomogram in validation set (mean absolute error = 0.007). (D) AUC of nomogram in validation set was 0.707 (95% CI, 0.657–0.758). AUC = area under curve.

Though obviously overestimated when probability was more than 30% in training set, bias-corrected calibration plot of the nomogram predicted LNM corresponding closely with the actual probability in both datasets. The mean absolute error in training set and validation set was 0.021 and 0.007, respectively. The AUC was 0.801 (95% CI, 0.729–0.873) in training set, and 0.707 (95% CI, 0.657–0.758) in validation set (Fig. 2).

### 3.4. A treatment algorithm for early gastric SRC

**Figure 3:**
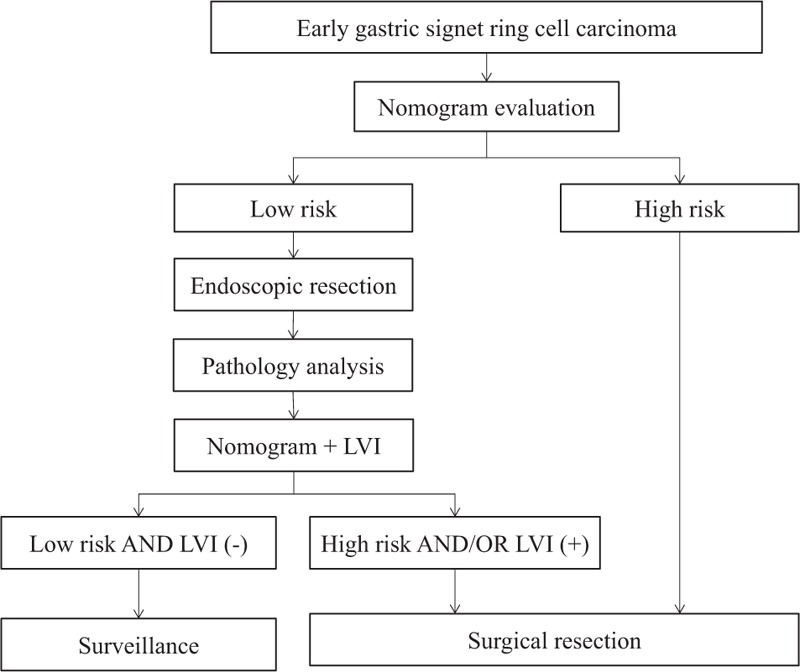
A treatment algorithm for patients with early gastric cancer with signet ring cell carcinoma.

**Figure 4:**
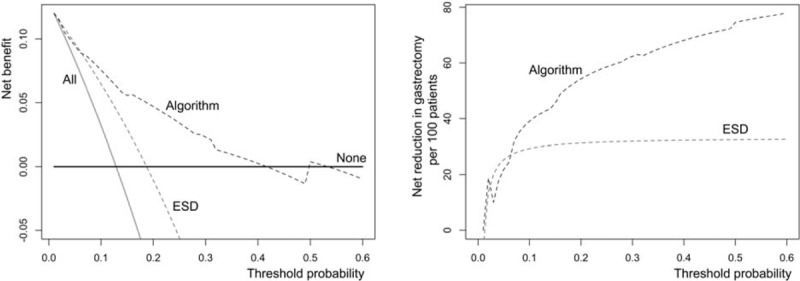
Clinical performance of the treatment algorithm for early gastric cancer with signet ring cell carcinoma. (A) The *y*-axis represents net benefits, calculated by subtracting the relative harms (false positives) from the benefits (true positives). The *x*-axis measures the threshold probability. A treatment strategy is superior if it has the highest value among models, including 2 simple strategies, such as performing surgery for all patients (sloping solid line) or no patients (horizontal solid line). For example, the value of net benefits would be 0.076 when 10% was selected as cutoff value, which means that nomogram would find about 7 patients with lymph node metastasis among one hundred patients compared with simple observation, without adding any unnecessary resections (false positives) theoretically. (B) The *y*-axis represents quantified reduction in gastrectomy, which means the net benefits without missing cancer patients (false negative) in theory.

Based on the nomogram, we constructed a treatment algorithm for patients with early gastric SRC as illustrated in Fig. 3. The threshold of risk stratification could be chosen at the preference of clinicians. Those evaluated as low-risk before ESD would be scrutinized again based on pathological analysis. Only those patients who met both low risk and negative LVI, were suggested a regular surveillance. In decision analysis of curve described as Vickers et al,^[18]^ our algorithm showed superiority to the current strategy (mucosal SRC without LVI, and size ≤2 cm) in most range (Fig. 4A). If 10% was arbitrary chose as a cutoff, 140 patients would be regarded as low risk in accordance with the final pathology analysis. Incidence of LNM was 2.9% (4/140) in the low-risk subgroup, and 25.0% (29/116) in the high-risk subgroup. Moreover, about 39 patients will spare an unnecessary resection without missing cancers compared with the strategy that resection on all patients in theory (Fig. 4B).

## 4. Discussion

In the present study, a nomogram predicting LNM in early gastric SRC was successfully established and validated in a large external dataset. Furthermore, a treatment algorithm was proposed to individual patients with SRC histology. These findings demonstrated that ESD could be applied to early gastric SRC under certain conditions. We believe this is a reliable prediction model and is useful for clinical counseling.

Several variables associated with LNM have been reported previously, including sex,^[20]^ depth of invasion,^[7]^ macroscopic type,^[8]^ tumor size,^[9]^ and LVI.^[6]^ In both this cohort and Japanese series, SRC histology tends to spread more superficially instead of invading deeply. Although there was a rich supply of small vessels in gastric mucosa, lymph capillaries were only distributed in the deeper lamina propria and submucosa,^[21]^ which may partly explained the low incidence of LNM in early gastric SRC. Interestingly, female sex was found to be involved with LNM in our findings, though female patients were frequently observed in younger patients^[22]^ or SRC,^[10,23]^ the role of estrogen hormone in gastric carcinogenesis remains unclear.

The performance of a prediction model should be validated in clinical practice. Though an obvious deviation appeared in the training set when predictive risk was over 30%, the predictive model has a good fitness to the actual probability in the external dataset, which was hence confirmed by a good concordance index. In the view of clinicians, clinical performance of a model is more valuable than discrimination ability, such as false negative and false positive. Therefore, decision analysis of curve was performed to quantify different strategies and determine an optimal threshold range. The findings demonstrated our algorithm was superior to the current indication for ESD (mucosal SRC without LVI and size ≤2 cm). Consequently, we proposed this treatment algorithm as ESD indication for patients with early gastric SRC. Different from previous studies,^[6–8,10]^ the highlight of the treatment algorithm is to provide a quantified risk score for individual counseling before ESD. In clinical practice, surgeons were encouraged to discuss with patients to determine a satisfied risk threshold. To the best of our knowledge, this is a first nomogram to predict LNM for early gastric SRC, which would help clinicians to balance quality of life and the aggressive resection.

Quantified risk evaluation maybe changes the design of treatment strategy. Sentinel lymph node biopsy (SLNB) was a promising detective procedure for LNM with sensitivity varying from 40% to 100%.^[24]^ Despite of a reported sensitivity of 87.8% in meta-analysis, SLNB may not be clinically applied due to high false negative and heterogeneity among studies.^[25]^ However, identifying patients with specified risk was probably alternative for application of SLNB. In clinical practice, we may be accepted a strategy that misdiagnosed a small number of patients by applying a procedure with a high false negative rate to a population with a controlled prevalence. Supposing SLNB would miss 10% patients. We would only miss 2 patients in a population with average risk of 20%. Ninety-eight patients will spare from excessive resection. Based on a reliable nomogram, individualized risk stratification would be applicable and allow a tailored therapy.

There are limitations in the retrospective study. First, inter pathologists bias possibly deviated the histologic diagnosis during a long period. SRC was defined as more than 50% of the tumor consisting of malignant cells containing intracytoplasmic mucin,^[26]^ which was easily categorized as poorly differentiated type and diagnostic threshold varied among pathologists. Though reviewed by 2 advanced pathologists in our institute, the diagnostic standard was unable to keep consistency with Japanese series. Therefore, a central pathology consensus of specimen was indicated in further research. Second, there was only a small sample size in the present study. However, a good diagnostic ability of the model was shown through validating in a large external dataset. Superior clinical utility to the current indication for ESD was also confirmed in quantified decision analysis. Thus, we thought this is a reliable prediction model. Finally, it was noted that the nomogram should be applied with caution. Zheng et al^[27]^ first reported a nomogram for predicting the incidence of LNM for submucosal gastric cancer. Based on a dataset of 262 patients, a nomogram was developed and validated internally with a discrimination power of 0.844. Later, they established a predicting nomogram for EGC using the same method.^[28]^ As serum tumor markers were associated with LNM, Zhao et al^[29]^ improved the diagnostic ability of the model by adding preoperative tumor markers, such as CEA, CA125 and CA19-9. These findings provided a quantified LNM risk for individual patients with EGC. However, most factors included in these papers and ours were pathological variables obtained after surgery, which could lead to evaluation bias before treatment. Besides, discrepancy was frequently observed in histologic diagnosis between biopsy and postoperative specimen.^[30]^ Herein, we provided a 2-step algorithm including pre- and postoperative evaluation to minimized the error.

In summary, based on large datasets from 2 high-volume institutions, a reliable nomogram for predicting LNM in patients with early gastric SRC was established and validated. Subsequently, an instructive protocol assisting clinicians in treatment was proposed and assessed. This novel treatment algorithm would be helpful to decision-making for patients with early gastric SRC.

## Acknowledgments

We gratefully thank Professor Takeshi Sano at the Cancer Institute Ariake Hospital, Tokyo, in Japan for his data sharing.
